# Robotic Versus Laparoscopic Anatomic Liver Resection: Comparison of Perioperative Outcomes—A Systematic Review and Meta‐Analysis

**DOI:** 10.1002/ags3.70183

**Published:** 2026-01-28

**Authors:** Yutaro Kato, Atsushi Sugioka, Hiroyuki Kato, Akihiko Horiguchi

**Affiliations:** ^1^ Department of Gastroenterological Surgery Fujita Health University Bantane Hospital Nagoya Japan; ^2^ International Medical Center Fujita Health University Hospital Toyoake Japan

**Keywords:** anatomic liver resection, laparoscopic liver resection, major hepatectomy, parenchyma‐sparing liver resection, robotic liver resection

## Abstract

**Aim:**

Minimally invasive anatomic liver resection (AR) is technically demanding, and the efficacy of robotic surgery in AR remains unestablished. This systematic review aims through a meta‐analysis to compare surgical outcomes between robotic (RAR) and conventional laparoscopic (LAR) AR.

**Methods:**

A systematic literature search of relevant studies published between 2001 and 2024 in PubMed/MEDLINE, Embase and Cochrane Library was carried out, and 15 studies were selected. Meta‐analysis was performed to compare perioperative outcomes between RAR and LAR.

**Results:**

A total of 4171 patients comprising 2042 RAR and 2129 LAR patients who underwent major hepatectomy or liver parenchyma‐sparing AR (PSAR) were included. All included studies were retrospective comparative studies, including eight using propensity score‐matched analysis. Meta‐analysis demonstrated that as primary outcomes, the 30‐day and 90‐day mortalities and postoperative overall morbidity were comparable between RAR and LAR, while RAR had significantly less morbidity≥Clavien‐Dindo grade II and a lower rate of open conversion. As secondary outcomes, compared to LAR, RAR showed significantly less blood loss and shorter postoperative hospital stay, while RAR had a higher rate of postoperative 30‐day readmission. Operative time, blood transfusion, Pringle maneuver, R0 resection, and reoperation were comparable. Subgroup meta‐analyses showed a lower rate of blood transfusion in robotic PSAR and a lower rate of open conversion in RAR in the right cranial regions.

**Conclusion:**

This large‐scale meta‐analysis of minimally invasive AR suggests that RAR can confer comparable or partly better perioperative outcomes as compared to LAR, indicating potential advantages of the robotic approach to AR.

## Graphical abstract

Minimally invasive anatomic liver resection (AR) including major hepatectomy and liver parenchyma‐sparing AR is technically complex and demanding. This systematic review with meta‐analysis including 15 studies comparing 2042 robotic AR and 2129 laparoscopic AR patients demonstrated largely comparable perioperative outcomes and partly better outcomes including significantly less major postoperative morbidity, a lower rate of open conversion, less blood loss, and shorter postoperative hospital stay in robotic AR. These results suggest potential perioperative advantages conferred by robotic surgery over laparoscopic surgery for this type of complex liver resection.

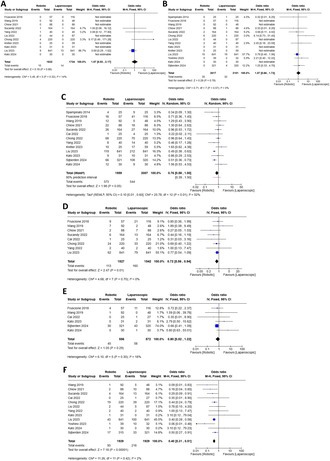

## 1 Introduction

Anatomic liver resection (AR) is a hepatectomy procedure for accurately resecting a liver territory supplied by the corresponding Glissonean pedicles (GPs), and AR includes not only major hepatectomy (MH) but also liver parenchyma‐sparing AR (PSAR). The PSAR is intended to achieve both high curability and functional safety particularly in resection of hepatocellular carcinoma [1, 2, 3]. The AR is technically demanding due to the requirements to accurately control the target GPs, to optimally resect the right amount of relevant liver parenchyma, and to expose the landmark or intersegmental hepatic veins [4, 5, 6].

Worldwide spread of minimally invasive liver resection (MILR) over the last three decades have increased the use of laparoscopy to AR mostly by expert surgeons [7, 8], although they can still recognize technical limitations in laparoscopic AR (LAR) in difficult situations. In this context, the advent and growing application of surgery‐assisting robots have increasingly enabled surgeons to perform MILR more safely and with less complications as compared to laparoscopic hepatectomy [9, 10]. This meta‐analysis investigates the impacts of robotic surgery on AR by comparing perioperative outcomes with conventional LAR.

## 2 Materials and Methods

### 2.1 Literature Search

The search strategy was based on the Preferred Reporting Items for Systematic Reviews and Meta‐Analyses (PRISMA) 2020 guideline [11]. We searched the English‐language full text articles on human studies published between Jan 2001 and Dec 2024 in the databases: MEDLINE (PubMed), Embase and Cochrane Library, using the following terms: (Robotic OR robotic‐assisted OR robotic‐assisted laparoscopic OR minimally invasive) AND (laparoscopic OR laparoscopic‐assisted OR laparoscopy) AND (liver resection OR hepatectomy OR hepatic resection OR hepatic surgery OR anatomic liver resection OR anatomic hepatectomy OR anatomical liver resection OR anatomical hepatectomy OR major liver resection OR major hepatectomy OR hemi‐hepatectomy OR right posterior sectionectomy OR right anterior sectionectomy OR central hepatectomy OR central bi‐sectionectomy OR central bi‐segmentectomy OR segmentectomy OR mono‐segmentectomy OR bi‐segmentectomy OR parenchymal‐sparing hepatectomy) AND (comparative study OR observational study OR randomized controlled study OR prospective study OR retrospective study).

### 2.2 Inclusion and Exclusion Criteria

The AR was defined to include: (1) MH to resect three or more contiguous Couinaud's segments; (2) (extended) right anterior sectionectomy (RAS), right posterior sectionectomy (RPS) and left medial sectionectomy (LMS); and (3) (extended) mono‐ or sub‐ segmentectomy, according to the Brisbane [12] and Tokyo 2020 [13] terminologies. The published studies on robotic AR (RAR) and LAR were screened, and eligible articles were selected according to the following inclusion and exclusion criteria. The inclusion criteria were as follows: comparative studies between RAR and LAR; hepatectomy for liver neoplasms; and perioperative outcomes were reported. The exclusion criteria were as follows: experimental studies; case or technical reports; review articles; hand‐assisted laparoscopic surgery; left lateral sectionectomy; and studies where the number of cases in either RAR or LAR cohort was less than 25. The last exclusion criterion was applied to reduce the effect of the learning curve; the cut‐off number was based on the reported median number of cases required to surmount the learning curve of robotic and laparoscopic hepatectomy was 25 and 50, respectively [14].

### 2.3 Data Extraction

Two authors (Y.K. and H.K.) independently curated data from each included study. The following parameters were extracted: author, year and study characteristics including country, study period, surgery centers, study design, diseases, type of hepatectomy, and baseline data including age, sex, American Society of Anesthesiology (ASA) score, tumor size and multiplicity, tumor malignancy, the Iwate difficulty level for the hepatectomy procedure [15], history of previous hepatectomy, and the presence of cirrhosis.

### 2.4 Primary and Secondary Outcomes

The primary outcomes were the mortality within 30 and 90 days after surgery, postoperative morbidity and open conversion. The morbidity was evaluated according to the Clavien–Dindo complication grades [16] (overall, C–D ≥ II, and C–D ≥ IIIa). The secondary outcomes were operative time, intraoperative blood loss, intraoperative blood transfusion, application of Pringle maneuver, postoperative length of hospital stay (LOS), R0 resection, reoperation, and readmission within 30 days after surgery.

### 2.5 Assessment of Study Quality and the Risk of Bias

To assess the quality and risk of bias in the included studies, the New Castle Ottawa scale (NOS) [17] was used for cohort studies and the ROBINS‐1 [18] for randomized controlled studies where applicable. The maximum NOS score was 9 points, and studies with 7–9 scores were considered high quality.

### 2.6 Statistical Analysis

For continuous data, the mean difference (MD) with 95% confidence interval (CI) was calculated. The median and range or interquartile range were converted to the mean and standard deviation using the relevant mathematical models [19]. For dichotomous data, odds ratio (OR) with 95% CI was evaluated. Forest plots were used to assess the comparative risks between RAR versus LAR. The between‐study heterogeneity was evaluated using *I* ^2^ statistics, where *I* ^2^ ≤ 25%, 25 < *I* ^2^ ≤ 50%, 50 < *I* ^2^ ≤ 75% and *I* ^2^ > 75% were considered as low, moderate, high, and very high, respectively [20]. The random‐effects and fixed‐effects model was used for analyses with *I* ^2^ > 50% and *I* ^2^ ≤ 50%, respectively. The publication bias was visually assessed using funnel plots [21]. Review Manager (Cochrane Collaboration, https://revman.cochrane.org/info) was used for all analyses. *p*‐values < 0.05 was considered statistically significant.

## 3 Results

**FIGURE 1:**
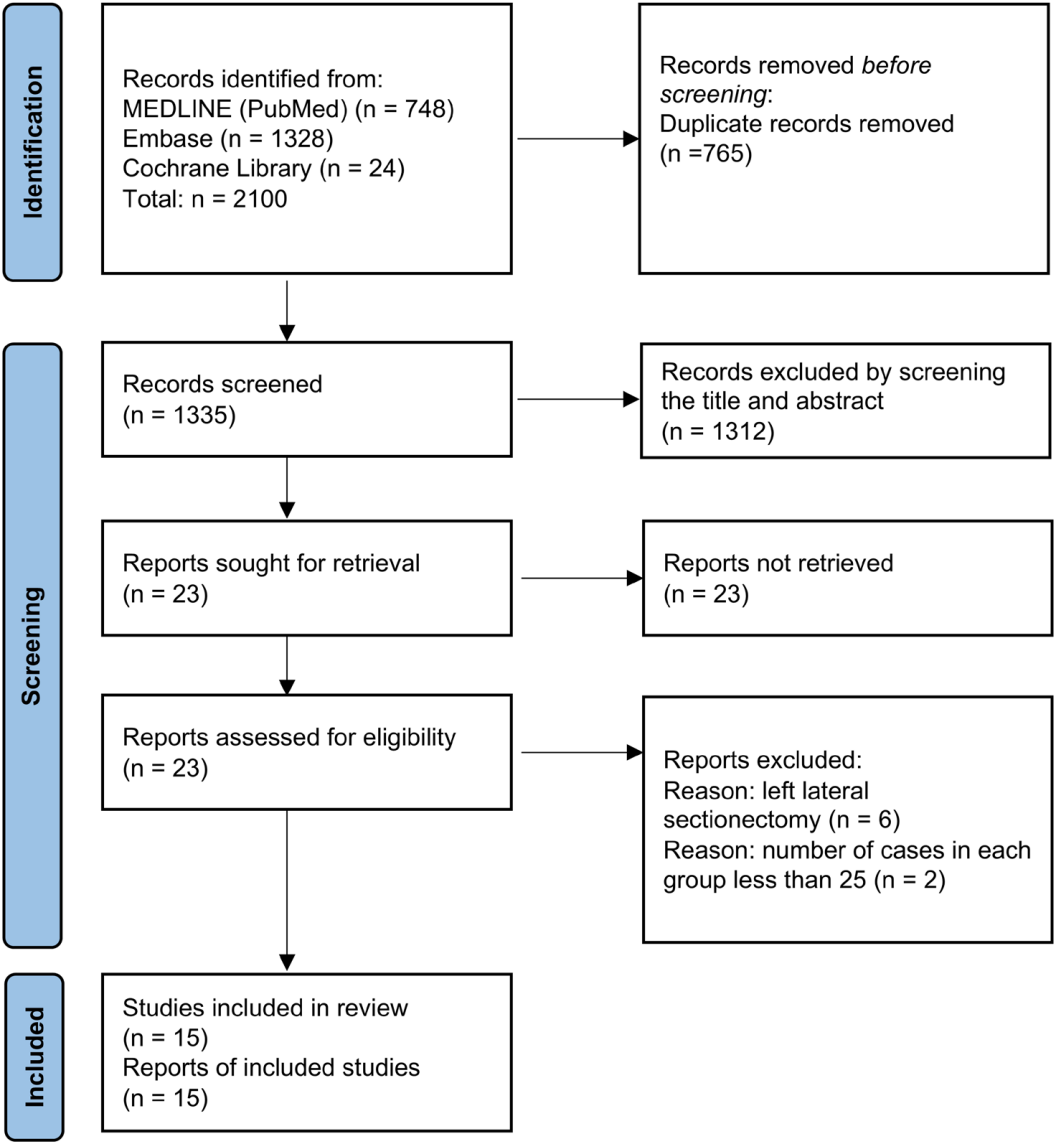
Flow diagram of study selection.

The flow diagram of study selection is shown in Figure 1. A total of 2100 articles were identified in the databases. After removal of duplicates and record screening based on the inclusion and exclusion criteria, we finally selected 15 studies for quantitative synthesis [4, 22, 23, 24, 25, 26, 27, 28, 29, 30, 31, 32, 33, 34, 35].

### 3.1 Quality Assessment

All included studies were retrospective cohort studies, and their quality was assessed using the NOS; all were of high quality (File S1).

### 3.2 Study Characteristics

The major characteristics of included studies are summarized in Table 1. They were published between 2014 and 2024, and the study periods ranged from 2008 to 2023. There were eight single‐center studies [4, 22, 24, 25, 26, 27, 31, 33] conducted in China (*n* = 3), USA (*n* = 2), Japan (*n* = 2), and Germany (*n* = 1), while there were one domestic [30] and six international multicenter studies [23, 28, 29, 32, 34, 35]. Two single‐center [4, 25] and six multicenter studies [23, 28, 29, 32, 34, 35] used propensity score matching (PSM) analyses. Types of AR were as follows: MH (*n* = 9) [22, 26, 27, 28, 29, 30, 31, 34, 35]; MH or RPS (*n* = 1) [33]; MH with partial resection (*n* = 1) [24]; MH, RPS, RAS, LMS, or segmentectomy (*n* = 1) [4]; RPS (*n* = 1) [23]; RAS or central hepatectomy (CH) (*n* = 1) [32]; and segmentectomy (*n* = 1) [25]. All studies included both malignant and benign diseases, except for two [4, 27] including hepatocellular carcinoma only.

**TABLE 1:** Summary of study characteristics.

Study, author	Publication year	Country	Study period	Study design	Disease	Type of AR
Cai [22]	2022	China	2015.4–2020.1	SC, Retro	Benign and malignant	MH
Chiow [23]	2021	International	2010–2019	MC, Retro, PSM	Benign and malignant	RPS
Chong [35]	2022	International	2008–2020	MC, Retro, PSM	Benign and malignant	MH
Fruscione [24]	2018	USA	2011–2016	SC, Retro	Benign and malignant	MH, MH+PR
Kato [4]	2023	Japan	2010–2022.10	SC, Retro, PSM	HCC, ICC, metastatic	MH, RPS, RAS, LMS, Seg
Kato [25]	2024	Japan	2010–2023.4	SC, Retro, PSM	HCC	Seg
Knitter [26]	2023	Germany	2017–2019	SC, Retro	Benign and malignant	MH
Liu [27]	2022	China	2017.1–2022.3	SC, Retro	HCC	MH
Liu [28]	2023	International	2008–2021	MC, Retro, PSM	Benign and malignant	MH
Sijberden [29]	2024	International	2009–2021	MC, Retro, PSM	Benign and malignant	MH
Spampinato [30]	2014	Italy	2009–2012	MC, Retro	Benign and malignant	MH
Sucandy [34]	2022	International	2008–2020	MC, Retro, PSM	Benign and malignant	MH
Wang [31]	2019	China	2011.11–2017.7	SC, Retro	Benign and malignant	MH
Yang [32]	2022	International	2010–2020	MC, Retro, PSM	Benign and malignant	RAS, CH
Yoshino [33]	2023	USA	2010–2021	SC, Retro	HCC, ICC, CRLM, others	MH, RPS

Abbreviations: AR, anatomic liver resection; CH, central hepatectomy; CRLM, colorectal metastasis; HCC, hepatocellular carcinoma; ICC, intrahepatic cholangiocarcinoma; LMS, left medial sectionectomy; MC, multicenter study; MH, anatomically major hepatectomy; PR, partial resection; PSM, propensity score matched analysis; RAS, right anterior sectionectomy; Retro, retrospective cohort study; RPS, right posterior sectionectomy; SC, single‐center study; Seg, (sub)segmentectomy.

The baseline patient and tumor characteristics are summarized in Table 2. A total of 4171 patients were included, comprising 2042 (49.0%) and 2019 in the RAR and LAR group, respectively. There were 1591 (38.1%) women comprising 805 (39.4%) in the RAR and 786 (36.9%) in the LAR group, respectively. The mean/median age ranged from 49.8 to 72.0 years and from 49.1 to 71.0 years in the RAR and LAR group, respectively. The reported ASA score was I or II in 2564 (64.3%) and III or IV in 1432 patients. The score was I or II in 1270 (64.3%) and 1294 (62.4%) patients and III or IV in 698 and 734 patients, in the RAR and LAR group, respectively. The mean/median tumor size ranged from 2.7 to 7.1 cm and 3.0 to 7.0 cm in the RAR and LAR group, respectively. Seven studies [4, 23, 25, 28, 32, 34, 35] reported the tumor multiplicity, which was 22.3% (316/1414; range: 14.6%–26.5%) and 19.6% (277/1414; range: 17.1%–30.0%) in the RAR and LAR group, respectively. The percentage of patients with malignant tumors was 81.5% (1633/2003; range: 48.0%–100%) and 81.4% (1707/2097; range: 46.6%–100%) in the RAR and LAR group, respectively. The Iwate difficulty level, which was reported in seven studies [4, 23, 25, 28, 32, 34, 35], was low, intermediate, advanced, and expert in 0 (0%), 33 (23.3%), 503 (35.6%), and 878 (62.1%) patients, and 0 (0%), 31 (21.9%), 521 (36.8%), and 862 (61.0%) patients, in the RAR and LAR group, respectively. History of previous hepatectomy, which was reported in eight studies [4, 23, 25, 28, 29, 32, 34, 35], was observed in 5.1% (88/1735; range: 2.3%–22.6%) and 4.6% (80/1735; range: 2.3%–23.3%) of patients in the RAR and LAR group, respectively. Cirrhosis was present in 24.5% (488/1992; range: 5.1%–61.4%) and 24.6% (504/2045; range: 3.1%–69.0%) of patients in the RAR and LAR group, respectively.

**TABLE 2:** Baseline characteristics of included patients.

Study, author	Procedure	Cases	Type of resection, *n* TS/(E)RH/(E)LH/CH+RAS/RPS/Seg/other	Age, years (R or IQR)	Sex, M/F, *n*	ASA I‐II/III‐IV	Tumor size, cm, (R or IQR)	Multiple tumors, *n* (%)	Malignant tumor, *n* (%)	Iwate level, L/I/A/E	Previous Hx, *n* (%)	Cirrhosis, *n* (%)
Cai [22]	RAR	25	0/0/25/0/0/0/0	56.4 ± 9.1^a^	12/13	2.1 ± 0.6^a^	5.5 ± 2.3^a^	N/A	12 (48.0)	N/A	N/A	7 (28.0)
	LAR	27	0/0/27/0/0/0/0	52.7 ± 11.6^a^	18/9	1.9 ± 0.6^a^	4.3 ± 1.9^a^	N/A	15 (55.6)	N/A	N/A	8 (29.6)
Chiow [23]	RAR	88	0/0/0/0/0/88/0	60 (51–69)	59/29	52/36	3.5 (3.0–5.0)	16 (18.2)	81 (92.0)	0/5/21/62	2 (2.3)	29 (33.0)
	LAR	88	0/0/0/0/0/88/0	61 (54–69)	64/24	56/31	4.0 (3.0–5.2)	17 (19.3)	83 (94.3)	0/2/23/63	2 (2.3)	32 (36.4)
Chong [35]	RAR	220	0/220/0/0/0/0/0	61.0 (51.9–69.0)	139/81	133/87	5.0 (3.0–7.0)	58 (26.4)	190 (86.4)	0/0/47/173	8 (3.6)	56 (25.5)
	LAR	220	0/220/0/0/0/0/0	61.0 (52.0–68.0)	148/72	133/87	4.7 (2.9–7.0)	48 (21.8)	190 (86.4)	0/1/49/170	12 (5.5)	25 (26.4)
Fruscione [24]	RAR	57	0/20/20/0/0/0/17	58.1 ± 15.7^a^	20/37	16/36	N/A	N/A	37 (64.9)	N/A	N/A	3 (5.3)
	LAR	116	0/46/22/0/0/0/48	53.2 ± 15.4^a^	52/64	30/72	N/A	N/A	54 (46.6)	N/A	N/A	7 (6.0)
Kato [4]	RAR	31	HH (4), Sec (7), Seg (20)	72 (21–82)	25/6	28/3	3.0 (1.2–12.5)	8 (25.8)	31 (100)	0/10/14/7	7 (22.6)	7 (22.6)
	LAR	31	HH (3), Sec (10), Seg (18)	70 (36–83)	25/6	29/2	3.0 (1.5–16.0)	8 (25.8)	31 (100)	0/5/18/8	6 (19.4)	6 (19.4)
Kato [25]	RAR	30	0/0/0/0/0/30/0	71 (63–74)	24/6	27/3	2.7 (2.0–3.5)	7 (23.3)	30 (100)	0/10/17/3	8 (26.7)	6 (20.0)
	LAR	30	0/0/0/0/0/30/0	71 (66–73)	26/4	29/1	3.0 (2.1–3.8)	9 (30.0)	30 (100)	0/8/20/2	7 (23.3)	11 (36.7)
Knitter [26]	RAR	25	0/9/16/0/0/0/0	61 (27–79)	9/16	16/9	N/A	N/A	17 (68)	N/A	N/A	N/A
	LAR	59	0/40/19/0/0/0/0	57 (26–83)	31/28	38/21	N/A	N/A	48 (81)	N/A	N/A	N/A
Liu [27]	RAR	44	0/13/27/4/0/0/0	49.82 ± 9.06^a^	29/15	N/A	≥ 5.0 (*n* = 20)	N/A	44 (100)	N/A	N/A	27 (61.4)
	LAR	87	0/43/30/14/0/0/0	51.49 ± 13.27^a^	61/26	N/A	≥ 5.0 (*n* = 29)	N/A	87 (100)	N/A	N/A	60 (69.0)
Liu [28]	RAR	841	0/304/294/70/173/0/0	61.0 (51.0–69.0)	504/333	530/311	5.0 (3.2–7.0)	186 (22.1)	715 (85.0)	0/0/269/572	43 (5.1)	221 (26.3)
	LAR	841	0/278/312/73/178/0/0	61.0 (52.0–70.0)	535/306	542/299	4.7 (3.0–7.0)	171 (20.3)	721 (85.7)	0/0/295/546	31 (3.7)	234 (27.8)
Sijberden [29]	RAR	321	TS (51), EHH (27), HH (232), CH (11)	62 (52–70)	195/126	220/101	5.3 (3.0–8.0)	N/A	229 (71.3)	N/A	12 (3.8)	66 (20.6)
	LAR	321	TS (46), EHH (29), HH (235), CH (11)	62 (50–72)	191/130	223/98	5.1 (2.8–8.2)	N/A	227 (70.7)	N/A	14 (4.4)	59 (18.4)
Spampinato [30]	RAR	25	0/17/7/0/0/1/0	63 (32–80)	13/12	22/3	N/A	N/A	17 (68)	N/A	N/A	N/A
	LAR	25	0/15/9/0/0/1/0	62 (33–80)	10/15	25/0	N/A	N/A	23 (92)	N/A	N/A	N/A
Sucandy [34]	RAR	164	0/0/164/0/0/0/0	62 (17.3)	100/64	104/60	4.1 (4.0)	24 (14.6)	131 (79.9)	0/8/127/29	5 (3.0)	26 (15.9)
	LAR	164	0/0/164/0/0/0/0	63 (15)	105/59	101/63	4.7 (3.0)	28 (17.1)	136 (82.9)	0/15/112/37	6 (5.5)	33 (20.1)
Wang [31]	RAR	92	0/44/48/0/0/0/0	54.1 ± 11.2^a^	55/37	88/4	7.1 ± 3.3^a^	N/A	61 (66.3)	N/A	N/A	20 (21.7)
	LAR	48	0/19/29/0/0/0/0	49.4 ± 13.0^a^	24/24	47/1	7.0 ± 3.3^a^	N/A	24 (50.0)	N/A	N/A	9 (18.8)
Yang [32]	RAR	40	0/0/0/0/40/0/0	62 (55–68)	32/8	29/11	3.8 (3.0–4.9)	7 (17.5)	38 (95.0)	0/0/8/32	3 (7.5)	18 (45.0)
	LAR	40	0/0/0/0/40/0/0	62 (54–72)	33/7	27/13	3.5 (3.0–5.0)	10 (25.0)	38 (95.0)	0/0/4/36	2 (5.0)	19 (47.5)
Yoshino [33]	RAR	39	13/15/0/0/1/10/0	70 (69–73)	17/22	5/34	5.3 (3.7–7.8)	N/A	N/A	N/A	N/A	2 (5.1)
	LAR	32	14/17/0/0/0/1/0	71 (68–74)	20/12	4/28	5.5 (4.4–7.7)	N/A	N/A	N/A	N/A	1 (3.1)

Abbreviations: (E)LH, (extended) left hemihepatectomy; (E)RH, (extended)right hemihepatectomy; ASA, American Society of Anesthesiology; CH, central hepatectomy; EHH, extended hemihepatectomy; HH, hemihepatectomy; Hx, hepatectomy; L/I/A/E, Low/Intermediate/Advanced/Expert; LAR, laparoscopic anatomic liver resection; N/A, not available; R or IQR, range or interquatile range; RAR, robotic anatomic liver resection; RAR/LAR, robotic/laparoscopic anatomic liver resection; RAS, right anterior sectionectomy; RPS, right posterior sectionectomy; Sec, sectionectomy; Seg, segmentectomy; TS, trisectionectomy.

^a^ Mean ± standard deviation.

### 3.3 Primary Outcomes

**FIGURE 2:**
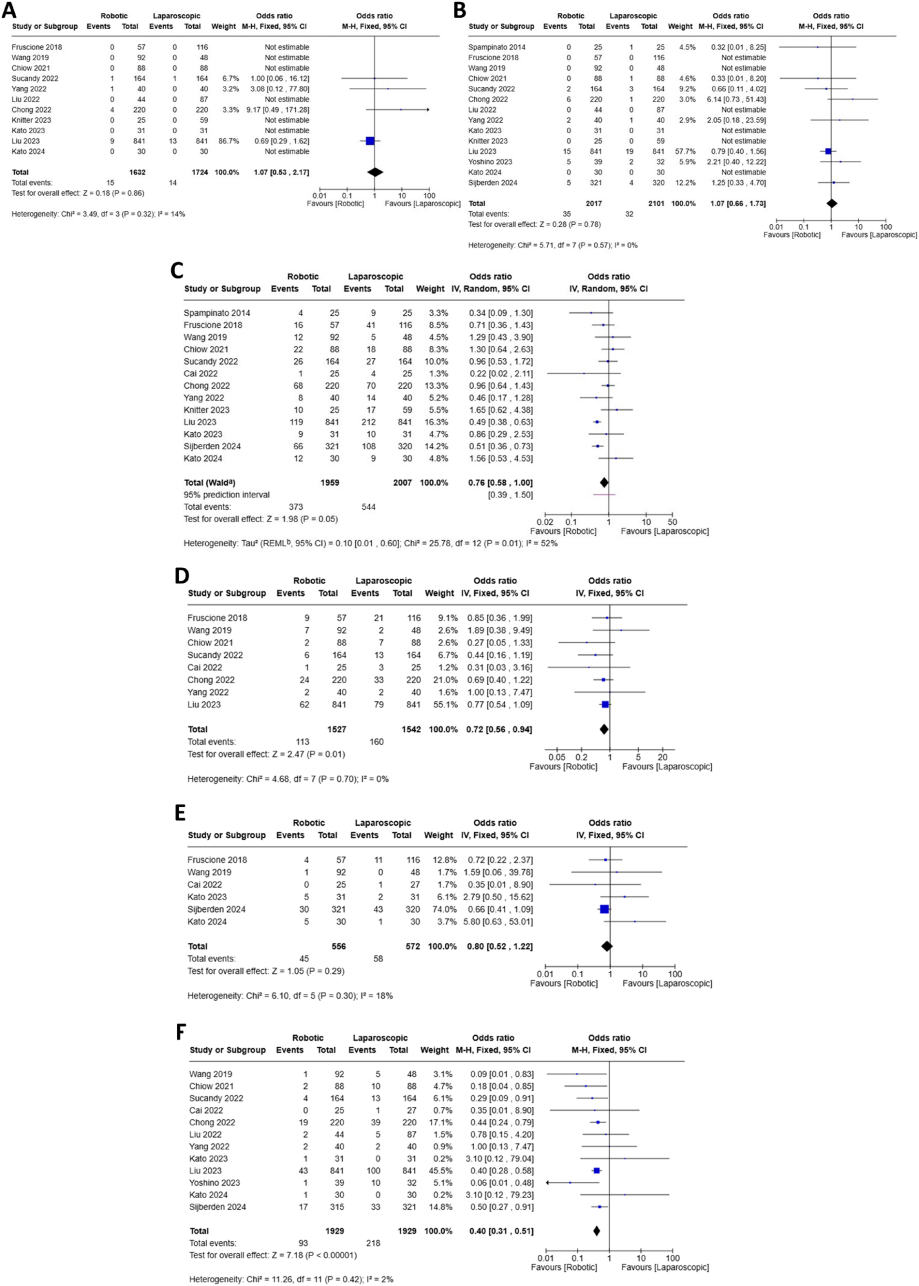
Forest plots of comparison between robotic anatomic liver resection (RAR) and laparoscopic anatomic liver resection (LAR) on primary outcomes: (A) 30‐day mortality, (B) 90‐day mortality, (C) overall postoperative morbidity, (D) postoperative morbidity of C–D ≥ II, (E) postoperative morbidity of C–D ≥ IIIa, (F) open conversion.

Eleven studies [4, 23, 24, 25, 26, 28, 31, 32, 34, 35] reported the postoperative 30‐day mortality rate, which was 0.9% (15/1632) and 0.8% (14/1724) in the RAR and LAR group, respectively. Meta‐analysis showed no between‐group difference in the 30‐day mortality (OR = 1.07, 95% CI = 0.53–2.17, *p* = 0.86), with a low between‐study heterogeneity (*p* = 0.32, *I* ^2^ = 14%) (Figure 2A). Fourteen studies [4, 23, 24, 25, 26, 27, 28, 29, 30, 31, 32, 33, 34, 35] reported the 90‐day mortality rate, which was 1.7% (35/2017) and 1.5% (32/2101) in the RAR and LAR group, respectively. Meta‐analysis showed no between‐group difference in the 90‐day mortality (OR = 1.07, 95% CI = 0.66–1.73, *p* = 0.78), with a low between‐study heterogeneity (*p* = 0.57, *I* ^2^ = 0%) (Figure 2B).

Thirteen [4, 22, 23, 24, 25, 26, 29, 30, 31, 32, 34, 35], eight [22, 23, 24, 28, 31, 32, 34, 35], and six [4, 22, 24, 25, 29, 35] studies reported overall, C–D ≥ II and C–D ≥ IIIa postoperative morbidity, respectively. The overall morbidity rate was 19.0% (373/1959) and 27.1% (544/2007) in the RAR and LAR group, respectively. Meta‐analysis demonstrated a marginally lower overall morbidity rate in the RAR group without statistical significance (OR = 0.76, 95% CI = 0.58–1.00, *p* = 0.05), but the between‐study heterogeneity was high (Figure 2C). The rate of morbidity of C–D ≥ II was 7.4% (113/1527) and 10.4% (160/1542) in the RAR and LAR group, respectively. Meta‐analysis showed a significantly lower rate of morbidity of C–D ≥ II in the RAR group (OR = 0.72, 95% CI = 0.56–0.94, *p* = 0.01), with a low between‐study heterogeneity (*p* = 0.70, *I* ^2^ = 0%) (Figure 2D). The rate of morbidity of C‐D ≥ IIIa was 8.1% (45/556) and 10.1% (58/572) in the RAR and LAR group, respectively. Meta‐analysis demonstrated no between‐group difference (OR = 0.83, 95% CI = 0.55–1.22, *p* = 0.29), with a low between‐study heterogeneity (*p* = 0.30, *I* ^2^ = 18%) (Figure 2E).

Twelve studies [4, 22, 23, 25, 27, 28, 29, 31, 32, 33, 34, 35] reported the rate of open conversion, which was 4.8% (93/1929) and 11.3% (218/1929) in the RAR and LAR group, respectively. Meta‐analysis demonstrated a significantly lower rate of open conversion in the RAR group (OR = 0.40, 95% CI = 0.31–0.51, *p* < 0.00001), with a low between‐study heterogeneity (*p* = 0.42, *I* ^2^ = 2%) (Figure 2F).

### 3.4 Secondary Outcomes

**FIGURE 3:**
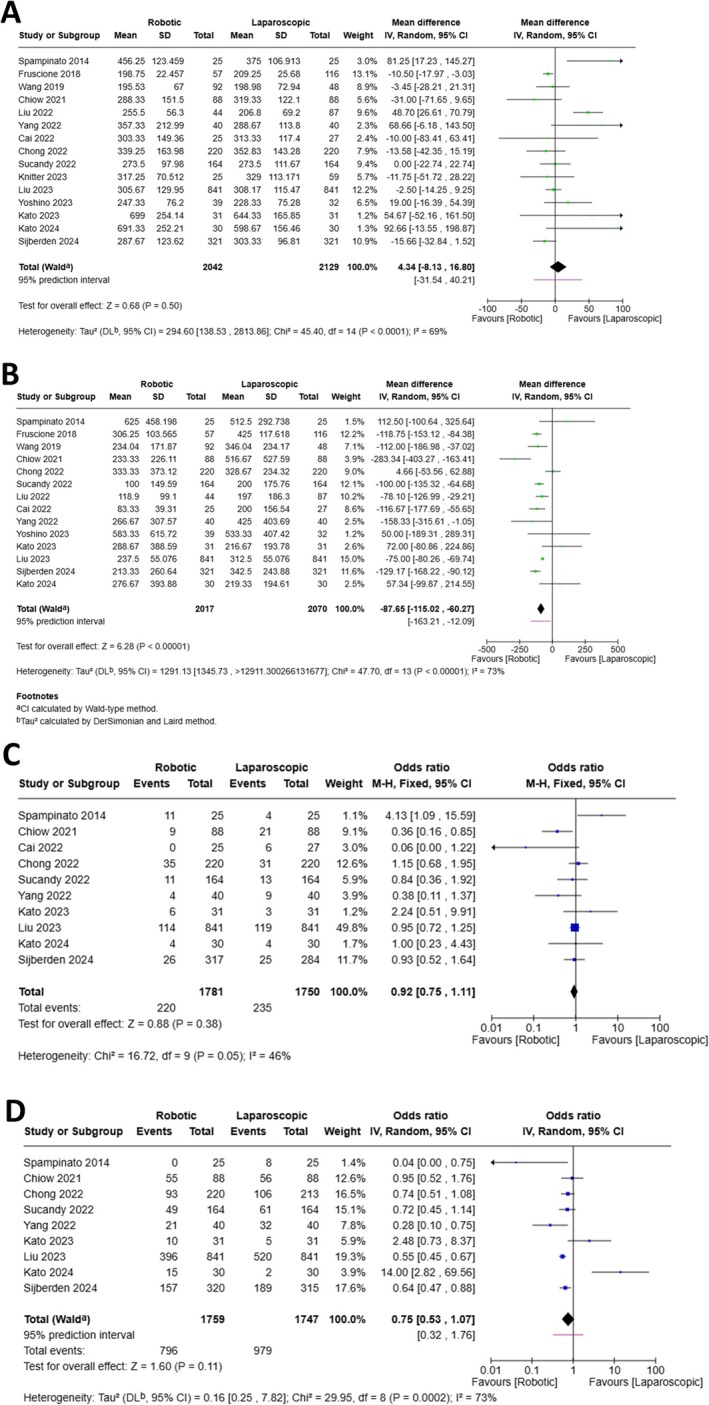
Forest plots of comparison between robotic anatomic liver resection (RAR) and laparoscopic anatomic liver resection (LAR) on secondary outcomes (Part 1): (A) operative time, (B) intraoperative blood loss, (C) intraoperative blood transfusion, (D) application of Pringle maneuver.

All included 15 studies [4, 22, 23, 24, 25, 26, 27, 28, 29, 30, 31, 32, 33, 34, 35] reported operative time. Meta‐analysis demonstrated comparable operative time between the RAR and LAR groups (MD = 4.34, 95% CI = −8.13–16.80, *p* = 0.50), with a high between‐study heterogeneity (*p* < 0.0001, *I* ^2^ = 69%) (Figure 3A). Fourteen studies [4, 22, 23, 24, 25, 27, 28, 29, 30, 31, 32, 33, 34, 35] reported intraoperative blood loss. Meta‐analysis showed significantly less intraoperative blood loss in the RAR group (MD = −87.65, 95% CI = −115.02–60.27, *p* < 0.00001), with a high between‐study heterogeneity (*p* < 0.00001, *I* ^2^ = 73%) (Figure 3B). Ten studies [4, 22, 23, 25, 28, 29, 30, 32, 34, 35] reported the rate of intraoperative blood transfusion, which was 12.4% (220/1781) and 13.4% (235/1750) in the RAR and LAR group, respectively. Meta‐analysis showed a comparable rate of intraoperative blood transfusion between the groups (OR = 0.92, 95% CI = 0.75–1.11, *p* = 0.38), with a moderate between‐study heterogeneity (*p* = 0.05, *I* ^2^ = 46%) (Figure 3C). Nine studies [4, 23, 25, 28, 29, 30, 32, 34, 35] reported the rate of application of Pringle maneuver, which was 42.5% (796/1759) and 56.0% (979/1747) in the RAR and LAR group, respectively. Meta‐analysis showed a comparable rate of application of Pringle maneuver between the groups (OR = 0.75, 95% CI = 0.53–1.07, *p* = 0.11), with a high between‐study heterogeneity (*p* = 0.0002, *I* ^2^ = 73%) (Figure 3D).

**FIGURE 4:**
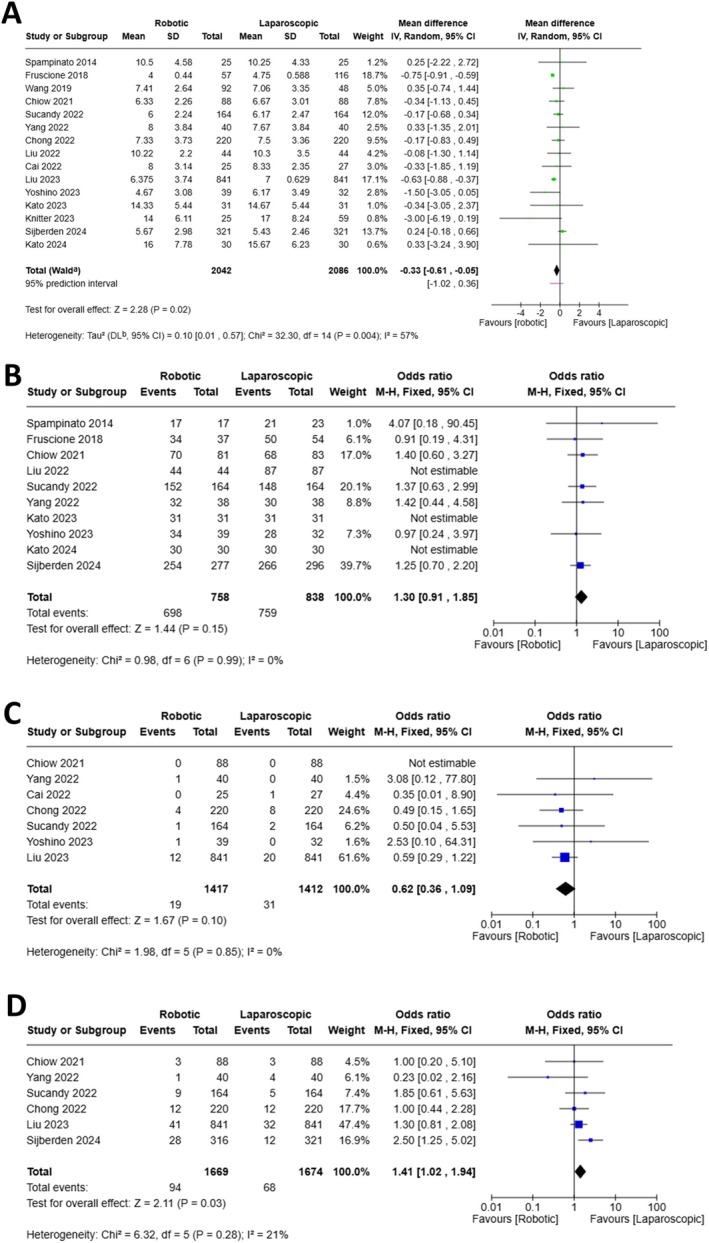
Forest plots of comparison between robotic anatomic liver resection (RAR) and laparoscopic anatomic liver resection (LAR) on secondary outcomes (Part 2): (A) length of postoperative hospital stay, (B) R0 resection, (C) Reoperation, (D) 30‐day readmission.

All included 15 studies [4, 22, 23, 24, 25, 26, 27, 28, 29, 30, 31, 32, 33, 34, 35] reported LOS. Meta‐analysis revealed significantly shorter LOS in the RAR group (MD = −0.33, 95% CI = −0.61–0.05, *p* = 0.02), with a high between‐study heterogeneity (*p* = 0.004, *I* ^2^ = 57%) (Figure 4A). Ten studies [4, 23, 24, 25, 27, 29, 30, 32, 33, 34] reported the R0 resection rate, which was 92.1% (698/758) and 90.6% (759/838) in the RAR and LAR group, respectively. Meta‐analysis showed a comparable R0 resection rate between the groups. (MD = 1.30, 95% CI = 0.91–1.85, *p* = 0.15), with a low between‐study heterogeneity (*p* = 0.99, *I* ^2^ = 0%) (Figure 4B). Seven studies [22, 23, 28, 32, 33, 34, 35] reported the incidence of reoperation, which was 1.3% (19/1417) and 2.2% (31/1412) in the RAR and LAR group, respectively. Meta‐analysis showed a comparable incidence of reoperation between the groups (OR = 0.62, 95% CI = 0.36–1.09, *p* = 0.10), with a low between‐study heterogeneity (*p* = 0.85, *I* ^2^ = 0%) (Figure 4C). Six studies [23, 28, 29, 32, 34, 35] reported the rate of 30‐day readmission, which was 5.6% (94/1669) and 4.1% (68/1674) in the RAR and LAR group, respectively. Meta‐analysis demonstrated a significantly higher 30‐day readmission rate in the RAR group (OR = 1.41, 95% CI = 1.02–1.94, *p* = 0.03), with a low between‐study heterogeneity (*p* = 0.28, *I* ^2^ = 21%) (Figure 4D).

### 3.5 Publication Bias

Funnel plot analyses (File S2) visually demonstrated that the publication bias of included studies was not obvious in the 30‐day mortality (S2A), 90‐day mortality (S2B), postoperative morbidity of C–D ≥ II (S2D) or C–D ≥ IIIa (S2E), open conversion (S2F), intraoperative blood transfusion (S2I), R0 resection (S2L), reoperation (S2M), and 30‐day readmission (S2N). Some publication biases were observed in overall morbidity (S2C), operative time (S2G), intraoperative blood loss (S2H), application of Pringle maneuver (S2J), and LOS (S2K).

### 3.6 Subgroup Analysis on Robotic and Laparoscopic PSAR

As a subtype of AR, PSAR was defined as (extended) RAS or RPS, (extended) central hepatectomy (CH) including central bi‐sectionectomy and LMS, or (extended) mono‐ or sub‐ segmentectomy. A subgroup meta‐analysis was conducted to compare perioperative outcomes between robotic (R‐PSAR) and laparoscopic (L‐PSAR) PSAR based on three studies [23, 25, 32] (Table 3).

**TABLE 3:** Summary of primary and secondary outcomes in robotic versus laparoscopic PSAR.

Outcomes	Studies (Ref. No.)	Data type	Number of patients	Events total *n* (%), (range, %)	OR or MD	*p*	Heterogeneity among studies	Heterogeneity level
			R‐PSAR/L‐PSAR	R‐PSAR, L‐PSAR	[95% CI]			
Primary outcomes								
30‐day mortality	[23, 25, 32]	Dichotomous	158/158	1 (0.6), (0–2.5) 0 (0), (0–0)	3.08 [0.12, 77.80]	0.50	N/A	N/A
90‐day mortality	[23, 25, 32]	Dichotomous	158/158	2 (1.3), (0–5.0) 2 (1.3), (0–2.5)	1.00 [0.17, 5.89]	1.00	*p* = 0.37, *I* ^2^ = 0%	Low
Overall morbidity	[23, 25, 32]	Dichotomous	158/158	42 (26.5), (25.0–40.0) 41 (25.9), (20.5–35.0)	1.01 [0.51, 2.00]	0.98	*p* = 0.19, *I* ^2^ = 40%	Moderate
Major morbidity ≥ C–D grade II	[22, 32]	Dichotomous	128/128	4 (3.1), (2.2–5.0) 9 (7.0), (5.0–8.0)	0.43 [0.13, 1.42]	0.17	*p* = 0.17, *I* ^2^ = 1%	Low
Open conversion	[23, 25, 32]	Dichotomous	158/158	5 (3.2), (2.3–5.0) 12 (7.6), (0–11.4)	0.42 [0.15, 1.18]	0.10	*p* = 0.10, *I* ^2^ = 39%	Moderate
Secondary outcomes								
Operative time	[23, 25, 32]	Continuous	158/158	N/A	34.38 [−49.59, 118.35]	0.42	*p* = 0.02, *I* ^2^ = 76%	Very high
Intraoperative blood loss	[23, 25, 32]	Continuous	158/158	N/A	−132.75 [−331.48, 65.99]	0.19	*p* = 0.003, *I* ^2^ = 82%	Very high
Intraoperative blood transfusion	[23, 25, 32]	Dichotomous	158/158	17 (10.8), (10.0–13.3) 34 (21.5), (13.3–23.9)	0.44 [0.24, 0.83]	0.01	*p* = 0.49, *I* ^2^ = 0%	Low
Length of hospital stay	[23, 25, 32]	Continuous	158/158	N/A	−0.20 [−0.90, 0.50]	0.58	*p* = 0.75, *I* ^2^ = 0%	Low
Pringle maneuver	[23, 25, 32]	Dichotomous	158/158	91 (57.6), (50.0–62.5) 90 (57.0), (6.7–80.0)	1.35 [0.25, 7.23]	0.73	*p* = 0.0002, *I* ^2^ = 88%	Very high
R0 resection	[23, 25, 32]	Dichotomous	149/151	132 (88.6), (84.2–100) 128 (84.8), (78.9–100)	1.41 [0.71, 12.80]	0.33	*p* = 0.99, *I* ^2^ = 0%	Low
Reoperation	[22, 32]	Dichotomous	128/128	1 (0.8), (0–2.5) 0 (0), (0–0)	3.08 [0.12, 77.80]	0.50	N/A	N/A
30‐day readmission	[22, 32]	Dichotomous	128/128	4 (3.1), (2.5–3.4) 7 (5.5), (3.4–10.0)	0.56 [0.16, 1.95]	0.36	*p* = 0.30, *I* ^2^ = 8%	Low

Abbreviations: C‐D, Clavien‐Dindo; CI, confidence interval [lower, upper]; Heterogeneity level: low: 0% < *I* ^2^ ≤ 25%; moderate: 25% < *I* ^2^ ≤ 50%; high: 50% < *I* ^2^ ≤ 75%; very high: *I* ^2^ > 75%; MD, mean difference (continuous data); N/A, not applicable; OR, odd's ratio (dichotomous data); PSAR, parenchyma‐sparing anatomic liver resection; R‐PSAR and L‐PSAR, robotic and laparoscopic PSAR, respectively.

As primary outcomes, meta‐analysis demonstrated no statistical differences in the 30‐day and 90‐day mortality, postoperative overall morbidity and morbidity of C‐D ≥ II, and open conversion between R‐PSAR and L‐PSAR.

As secondary outcomes, meta‐analysis showed no statistical differences in operative time, intraoperative blood loss, LOS, the application of Pringle maneuver, R0 resection, reoperation, and 30‐day readmission between R‐PSAR and L‐PSAR. The rate of intraoperative blood transfusion was 10.8% and 21.5% in R‐PSAR and L‐PSAR, respectively. Meta‐analysis demonstrated a significantly lower rate of intraoperative blood transfusion in the R‐PSAR group (OR = 0.44, 95% CI = 0.24–0.83, *p* = 0.01), with a low between‐study heterogeneity (*p* = 0.49, *I* ^2^ = 0%).

### 3.7 Subgroup Analysis on AR in the Right Hepatic Cranial Regions

A subgroup meta‐analysis on AR in the right hepatic cranial regions was performed to evaluate the potential advantage of robotic surgery in this demanding procedure. As details of resected segments such as segment 7 or 8 were unavailable, the meta‐analysis was based on three studies [23, 32, 35] analyzing RAS and CH [32], RPS [23] and (extended) right hemihepatectomy [35] (Table 4).

**TABLE 4:** Summary of primary and secondary outcomes in robotic versus laparoscopic AR in the cranial regions of the right liver.

Outcomes	Studies (Ref. No.)	Data type	Number of patients	Events total *n* (%), (range, %)	OR or MD	*p*	Heterogeneity among studies	Heterogeneity level
			RAR/LAR	RAR, LAR	[95% CI]			
Primary outcomes								
30‐day mortality	[23, 32, 35]	Dichotomous	348/348	5 (1.4), (0–1.8) 0 (0), (0–0)	5.60 [0.64, 49.03]	0.12	*p* = 0.62, *I* ^2^ = 0%	Low
90‐day mortality	[23, 32, 35]	Dichotomous	348/348	8 (2.3), (0–2.7) 2 (0.6), (0–2.5)	3.83 [0.77, 19.02]	0.10	*p* = 0.51, *I* ^2^ = 0%	Low
Overall morbidity	[23, 32, 35]	Dichotomous	348/348	98 (28.2), (20.0–30.9) 102 (29.3), (20.5–35.0)	0.95 [0.68, 1.32]	0.75	*p* = 0.26, *I* ^2^ = 25%	Low
Morbidity ≥ C–D grade II	[23, 32, 35]	Dichotomous	348/348	28 (8.0), (5.0–10.9) 40 (12.1), (5.0–15.0)	0.64 [0.39, 1.08]	0.09	*p* = 0.50, *I* ^2^ = 0%	Low
Open conversion	[23, 32, 35]	Dichotomous	348/348	23 (6.6), (2.6–8.6) 51 (14.7), (5.0–17.7)	0.41 [0.24, 0.69]	0.0007	*p* = 0.39, *I* ^2^ = 0%	Low
Secondary outcomes								
Operative time	[23, 32, 35]	Continuous	348/348	N/A	−3.56 [−45.20, 38.08]	0.87	*p* = 0.07, *I* ^2^ = 62%	Very high
Intraoperative blood loss	[23, 32, 35]	Continuous	348/348	N/A	−140.08 [−335.34, 55.18]	0.16	*p* < 0.0001, *I* ^2^ = 90%	Very high
Intraoperative blood transfusion	[23, 32, 35]	Dichotomous	348/348	48 (13.8), (10.0–15.9) 61 (17.5), (14.1–23.9)	0.75 [0.50, 1.14]	0.18	*p* = 0.04, *I* ^2^ = 69%	Very high
Length of hospital stay	[23, 32, 35]	Continuous	348/348	N/A	−0.19 [−0.68, 0.29]	0.44	*p* = 0.77, *I* ^2^ = 0%	Low
Pringle maneuver	[23, 32, 35]	Dichotomous	348/348	169 (48.6), (42.3–62.5) 194 (55.7), (48.2–80.0)	0.74 [0.55, 1.01]	0.05	*p* = 0.10, *I* ^2^ = 56%	High
R0 resection	[23, 32]	Dichotomous	119/121	102 (85.7), (84.2–86.4) 98 (81.0), (78.9–81.9)	1.41 [0.71, 2.80]	0.33	*p* = 0.99, *I* ^2^ = 0%	Low
Reoperation	[23, 32, 35]	Dichotomous	348/348	5 (2.3), (0–2.5) 8 (2.3), (0–3.6)	0.64 [0.22, 1.89]	0.42	*p* = 0.30, *I* ^2^ = 8%	Low
30‐day readmission	[23, 32, 35]	Dichotomous	348/348	16 (4.6), (2.5–5.5) 15 (4.3), (0–5.5)	1.07 [0.52, 2.17]	0.86	*p* = 0.80, *I* ^2^ = 0%	Low

Abbreviations: C–D, Clavien–Dindo; CI, confidence interval [lower, upper]; Heterogeneity level, low, 0% < *I* ^2^ ≤ 25%; moderate, 25% < *I* ^2^ ≤ 50%; high, 50% < *I* ^2^ ≤ 75%; MD, mean difference (continuous data); N/A, not applicable; OR, odd's ratio (dichotomous data); RAR and LAR, robotic and laparoscopic AR, respectively; very high, *I* ^2^ > 75%.

As primary outcomes, no statistical between‐group differences were observed in 30‐day and 90‐day mortality, postoperative overall morbidity and morbidity of C‐D ≥ II, while the conversion rate was significantly lower in RAR (OR = 0.41, 95% CI = 0.24–0.69, *p* = 0.0007), with a low between‐study heterogeneity (*p* = 0.39, *I* ^2^ = 0%). As secondary outcomes, the meta‐analysis showed no between‐group differences in operative time, intraoperative blood loss and transfusion, LOS, R0 resection, reoperation, and 30‐day readmission.

## 4 Discussion

This meta‐analysis based on 15 studies including 4171 patients compared the perioperative outcomes between RAR and LAR. This study may have some strengths. First, a relatively large number of patients, more than 2000 in each RAR or LAR group, were included. Second, only studies with no less than 25 cases in each group were included to potentially mitigate the bias related to the learning curve. Third, eight of the 15 studies covering 3520 patients (84.3%) used PSM analysis, which could have increased the study reliability. Forth, as a subgroup meta‐analysis, R‐PSAR and L‐PSAR were compared for the first time to date. Finally, an additional subgroup meta‐analysis was performed for AR in technically demanding right hepatic cranial regions.

Our meta‐analysis demonstrated that compared to LAR, RAR had lower postoperative morbidity of C‐D ≥ II, less intraoperative blood loss, a lower rate of open conversion, and shorter LOS. The 30‐day and 90‐day mortality, overall morbidity, morbidity of C‐D ≥ IIIa, operative time, and rates of blood transfusion, R0 resection and reoperation were comparable. These results suggest that RAR can largely offer comparable or partly better perioperative outcomes as compared to LAR. The improvements in postoperative morbidity, blood loss, and the open conversion rate in RAR could be explained by dexterous and precise surgery using robotics in complex hepatectomy such as AR. Furthermore, shorter LOS in RAR might result from reduced significant complications and open conversion. On the other hand, the current meta‐analysis showed that RAR had a higher rate of 30‐day readmission, and a similar observation was reported in a recent large meta‐analysis comparing major and minor robotic versus laparoscopic liver resection [36]. Although the reasons for a higher readmission rate in RAR are unclear, the recent promotion of enhanced postsurgical recovery protocols might be related, because it leads to shorter LOS, consequently increasing a probability that latent complications become evident after discharge.

Previous meta‐analyses have compared robotic and laparoscopic hepatectomy in major resections [37, 38, 39, 40], major and minor resections or unknown type of resections [9, 36, 41, 42, 43, 44, 45]. Such a variety of included hepatectomy type may hinder proper comparison of outcomes. On the other hand, several meta‐analyses have specifically compared robotic (RMH) and laparoscopic (LMH) MH or have conducted subgroup analyses on MH. The largest meta‐analysis on robotic versus laparoscopic liver resection to date by Pilz da Cunha et al. [36], which included a subgroup of 3319 MH patients (1649 RMH, 1670 LMH), demonstrated that RMH was associated with a lower open conversion rate and a decreased risk for morbidity of C‐D ≥ IIIa, while RMH and LMH had comparable blood loss and LOS. In a meta‐analysis by Mao et al. [39] including 12 studies comprising 796 RMH and 861 LMH patients, RMH demonstrated a lower rates of morbidity of C‐D ≥ IIIa and open conversion, less blood loss, and shorter LOS. A meta‐analysis by Ziogas et al. [40] largely showed comparable perioperative outcomes between RMH (*n* = 225) and LMH (*n* = 300). A meta‐analysis by Coletta et al. based on eight studies comparing 244 robotic and 241 laparoscopic hemihepatectomy patients [38] showed a significantly lower open conversion rate, less blood loss and longer LOS in the robotic group, while the rates of blood transfusion, reintervention and R0 resection, morbidity and mortality were comparable. In a subgroup meta‐analysis of MH by Ciria et al. [42], RMH had more blood loss, a higher rate of transfusion and a lower rate of open conversion. A subgroup meta‐analysis of MH by Wang et al. [37] found reduced blood loss and decreased rates of open conversion and postoperative major morbidity in RMH. Obviously, contradictory results are found between the meta‐analyses, which may derive from differences in the study selection strategy and the inclusion and exclusion criteria. Nonetheless, the previous and our meta‐analyses have almost consistently demonstrated decreased rates of open conversion and postoperative morbidity in RMH or RAR, while the advantage of robotic surgery in other perioperative outcomes remains uncertain in MH or AR.

Few studies included in our meta‐analysis reported details of postoperative complications after RAR or LAR. In a study on left hemihepatectomy by Cai et al. [22], the robotic group had one patient complicated by bile leak (CD‐II), whereas 4 of the 27 laparoscopic patients had complications, including ascites (CD‐II, *n* = 2), bile leak (CD‐II, *n* = 1), and intraabdominal bleeding (CD‐III, *n* = 1). An MH study by Spampinato et al. [30] reported that compared to the laparoscopic group, the robotic group tended to be less complicated by bile leak (4% vs. 8%), transient liver failure (0% vs. 8%) and portal vein thrombosis (0% vs. 4%), without significant statistical differences.

In this meta‐analysis, we specifically compared RAR and LAR primarily because we presumed that robotics could surpass laparoscopic techniques utilizing its known functional advantages particularly in complex hepatectomy represented by AR. We also hypothesized that PSAR, which should be technically more complex than straightforward hemihepatectomy, can receive benefits from robotics particularly when the extrahepatic Glissonean approach is applied [4, 25]. However, our results based on three studies [23, 25, 32] did not demonstrate positive perioperative advantages of R‐PSAR over L‐PSAR, except for a reduced rate of blood transfusion. Additionally, we performed a subgroup analysis on AR of the right hepatic cranial regions to evaluate technical advantages of robotic surgery in this demanding procedure. Our results showing a significantly lower risk for conversion may suggest a potential advantage of robotics in this particular surgical setting, in line with previous studies demonstrating a lower conversion rate in robotic posterosuperior minor resection [36, 46]. However, the small number of studies and patients included in our meta‐analysis precludes conclusions, requiring larger meta‐analyses.

An important issue in evaluating the efficacy of robotic liver resection is medical cost. Our meta‐analysis did not assess this aspect because of limited data. Although robotic resection has been shown to be more costly than laparoscopic [42, 44, 45], shorter operative time, reduced blood transfusion, lower postoperative morbidity, or shorter LOS in robotic resection shown in recent studies [28, 29, 35] and this meta‐analysis in addition to decreased total labor cost for robotic solo‐surgery would lead to reduced overall cost for MILR by using robots.

This meta‐analysis has several limitations. First, all included studies are non‐randomized retrospective studies. Second, there were large differences in sample size among included studies. Third, high between‐study heterogeneities and unignorable publication biases were observed in some outcomes. Finally, PSAR accounted only for 7.6% (*n* = 316) of all included surgeries. Such a low proportion of PSAR patients may bias the evaluation of RAR, although technical advantages of robotics should be salient in PSAR.

## 5 Conclusion

Our meta‐analysis including a large number of patients suggests that RAR can confer comparable or partly better perioperative outcomes than LAR. Meta‐analyses including more patients and further hepatectomy‐type specific analyses are warranted to elucidate the advantages of the robotic approach to AR.

## Author Contributions

**Yutaro Kato:** conceptualization, methodology, data curation, investigation, formal analysis, writing – original draft, writing – review and editing, software, visualization, project administration. **Atsushi Sugioka:** conceptualization, supervision, writing – review and editing, validation. **Hiroyuki Kato:** data curation, writing – review and editing, validation, methodology, formal analysis, investigation. **Akihiko Horiguchi:** supervision, writing – review and editing.

## Funding

The authors have nothing to report.

## Ethics Statement

The authors have nothing to report.

## Consent

The authors have nothing to report.

## Conflicts of Interest

Akihiko Horiguchi and Hiroyuki Kato are editorial members of *Annals of Gastroenterological Surgery*. The other authors have nothing to report.

## Supporting information


**File S1.** New‐Ottawa scale scores of included studies.
**File S2.** Funnel plots on (A) 30‐day mortality, (B) 90‐day mortality, (C) overall postoperative morbidity, (D) postoperative morbidity of C‐D ≥ II, (E) postoperative morbidity of C‐D ≥ IIIa, (F) open conversion, (G) operative time, (H) intraoperative blood loss, (I) intraoperative blood transfusion, (J) application of Pringle maneuver (K) length of postoperative hospital stay, (L) R0 resection, (M) reoperation, and (N) 30‐day readmission.
**Table S1.** New‐Ottawa scale scores of included studies.
