# Supplementary material for: Robotic Versus Laparoscopic Anatomic Liver Resection: Comparison of Perioperative Outcomes—A Systematic Review and Meta‐Analysis
Source: Ann Gastroenterol Surg. 2026 Jan 28;10(4):960–74. doi: 10.1002/ags3.70183 (PMC13327094; doi:10.1002/ags3.70183)
Supplement: Supplementary file 1 — File S1. New‐Ottawa scale scores of included studies. File S2. Funnel plots on (A) 30‐day mortality, (B) 90‐day mortality, (C) overall postoperative morbidity, (D) postoperative morbidity of C‐D ≥ II, (E) postoperative morbidity of C‐D ≥ IIIa, (F) open conversion, (G) operative time, (H) intraoperative blood loss, (I) intraoperative blood transfusion, (J) application of Pringle maneuver (K) length of postoperative hospital stay, (L) R0 resection, (M) reoperation, and (N) 30‐day readmission. Table S1. New‐Ottawa scale scores of included studies. [file AGS3-10-960-s001.zip › ags370183-sup-0001-TableS1@Supplementary File S1.docx]

**Supplementary Table 1** New-Ottawa scale scores of included studies

| Author of studies  (Ref. No.) | Cai  22) | Chiow  23) | Chong  35) | Fruscione  24) | Kato  4) | Kato  25) | Knitter  26) | Liu L  27) | Liu Q  28) | Sijberden  29) | Spampinato  30) | Sucandy  34) | Wang  31) | Yang  32) | Yoshino  33) |
| --- | --- | --- | --- | --- | --- | --- | --- | --- | --- | --- | --- | --- | --- | --- | --- |
| Representative of the exposed cohort | 1 | 1 | 1 | 1 | 1 | 1 | 1 | 1 | 1 | 1 | 1 | 1 | 1 | 1 | 1 |
| Selection of the non external cohort | 1 | 1 | 1 | 1 | 1 | 1 | 1 | 1 | 1 | 1 | 1 | 1 | 1 | 1 | 1 |
| Ascertainment of exposure | 1 | 1 | 1 | 1 | 1 | 1 | 1 | 1 | 1 | 1 | 1 | 1 | 1 | 1 | 1 |
| Demonstration that outcome of interest was not present at start of study | 1 | 1 | 1 | 1 | 1 | 1 | 1 | 1 | 1 | 1 | 1 | 1 | 1 | 1 | 1 |
| Comparability of mortality | 1 | 1 | 1 | 1 | 1 | 1 | 1 | 1 | 1 | 1 | 1 | 1 | 1 | 1 | 1 |
| Comparability of disease (diagnosis) | 1 | 1 | 1 | 1 | 1 | 1 | 1 | 1 | 1 | 1 | 1 | 1 | 1 | 1 | 1 |
| Assessment of outcome | 1 | 1 | 1 | 1 | 1 | 1 | 1 | 1 | 1 | 1 | 1 | 1 | 1 | 1 | 1 |
| Enough time for follow-up (≥90 days) | 0 | 1 | 1 | 1 | 1 | 1 | 1 | 1 | 1 | 1 | 1 | 1 | 1 | 1 | 1 |
| Adequacy (≥90%)  of follow up | 0 | 0 | 0 | 0 | 1 | 1 | 1 | 0 | 0 | 0 | 0 | 0 | 0 | 0 | 0 |
| Evaluation  (total score) | 7 | 8 | 8 | 8 | 9 | 9 | 9 | 8 | 8 | 8 | 8 | 8 | 8 | 8 | 8 |
